# How to leverage implementation research for equity in global health

**DOI:** 10.1186/s41256-024-00388-5

**Published:** 2024-10-17

**Authors:** Olakunle Alonge

**Affiliations:** Sparkman Center for Global Health, UAB School of Public Health, The University of Alabama at Birmingham, 1665 University Blvd, 517C, Birmingham, AL 35233 USA

## Abstract

Implementation research (IR) is important for addressing equity in global health. However, there is limited knowledge on how to operationalize IR for health equity, and pathways for improving health equity through IR in global health settings. This paper provides an overview of guidance and frameworks for thinking about health equity as part of IR while noting the gaps in how this guidance and frameworks apply to global health. It proposes an approach to guide implementation teams in the application of IR for achieving equity in global health considering these gaps. It describes key equity considerations for different aspects of IR (i.e., implementation contexts, strategies, outcomes, and research designs). These considerations can be applied prospectively and retrospectively, and at different stages of IR. The paper further describes causal pathways, intervention levers, and strategies for achieving health equity in global health settings through IR. Central to these pathways is the power asymmetries among different actors involved in IR in global health and how these contribute to health inequities. The paper suggests recommendations and strategies for shifting the balance of power among these actors while addressing the structural and systemic determinants of health inequities as part of IR. Explicit considerations for health equity as part of implementation research and practice are needed for the achievement of global health goals. Such explicit considerations should look back as much as possible, and entail defining and analyzing health inequities and intervening on the underlying causes and mechanisms of health inequities as part of IR on a routine basis.

## Background 

Implementation of health interventions occurs within health systems – dynamic and complex arrangements that shape the distribution of health and related resources, structure, and power [1] – and impacts health equity, for better or worse. The complex and dynamic interactions within health systems may be more significant for implementation in low- and middle-income countries (LMIC) [2, 3], and may constrain implementation outcomes and their impact on health equity given the resource limitation in these settings [4]. Implementation of evidence-supported health interventions (ESI) – encompassing programs, policies, and individual practices [5] – do not occur in a vacuum. The adoption of evidence involves people, power, politics, and not just science [6, 7], which makes the impact of implementing ESI on health equity less predictable – especially in places where institutions, norms, and enforcements are less well-established.

Hence, there has been an increased call for implementation research (IR) to systematically address issues of health equity [8–11]. IR is sometimes interchangeably called implementation science (IS) or considered a sub-domain of IS applied to improving healthcare and health impact [12–14]. It is defined as “the scientific inquiry into questions concerning implementation, i.e., the act of carrying an intention (e.g., ESI) into effect” [5]. This encompasses the specification of implementation strategies that are fit for context and purpose, and explication of pathways and outcomes through which ESI and strategies produce impact in real-world settings [15]. Health equity is defined as “everyone having a fair and just opportunity to be as healthy as possible” [16]. Achieving health equity through IR requires insightful analyses of systems and policies in which implementation is taking place, as well as an understanding of the needs, culture and history of the peoples involved. [10, 17, 18].

However, there is limited guidance on how to use IR to address equity in global health [9–11], especially in LMIC settings [19]. Global health and health equity may be synonymous – to the extent that global health is defined as a field of study that places priority on improving health and achieving equity in health for all people worldwide [20], and emphasizes the needs of the most vulnerable populations in resource-limited settings using an interdisciplinary approach rooted in public health [21]. It is difficult to fulfill the aspirations of global health without implementing ESI in LMIC, and operationalizing IR to achieve equity in these settings [22]. The paucity of a systematic guidance on how IR can be leveraged for achieving equity in LMIC settings hampers the aspirations of global health.

To be clear, guidance exists on how IR can be prospectively applied (i.e., during the planning and implementation of an ESI or IR study) to better tackle health inequities [8–11, 19, 23]. However, few of these specifically target teams working to achieve global health in LMIC settings [19], or provide explicit guidance on how such considerations can be retrospectively applied (i.e., after the implementation of an ESI or IR study has been long completed). Such retrospective analyses are important for understanding how and why completed implementation projects or IR studies may have improved or worsened equity to generate learnings for future applications [24, 25]. Indeed, the implications of choices and decisions made around implementing an ESI on health equity may not be completely knowable at the outset, and only emerge with the passage of time – given the complex and adaptive nature of health systems [26]. Further, insights from retrospective analyses may be especially important in global health initiatives where historical antecedents governing power relations between entities in LMIC and high-income countries (HIC) may have dominated priority-setting around a health problem and ESI, and decisions on what, when, where, and how to implement [27, 28].

The history of colonization, slavery, and group supremacy has played a prominent role in shaping global health practices and relationships between HIC and LMIC [27, 28], and within certain countries [29, 30], and continues to be a major contributor to the extant health inequities globally [27–30]. The issue of decolonizing global health cannot be side-stepped in considering how IR can be leveraged for achieving equity in LMIC [27–31], and it is nearly impossible to address this issue without looking back as far as possible to see how history may have shaped a given health system and the implementation of ESI within that system. For this issue and other historical antecedents, guidance on how to operationalize IR not only prospectively, but also retrospectively may be needed to achieve health equity.

## Development of a systematic approach for leveraging IR to address health equity in global health

This paper proposes a systematic approach to guide implementation teams in the application of IR for achieving equity in global health. It describes key considerations that can be incorporated into IR either prospectively and/or retrospectively to achieve equity and explicates pathways and strategies through which IR can be used to address health equity in global health. To develop the systematic approach, a synthesis of existing guidance on how to address health equity through IR, and gaps within that guidance, was first conducted drawing from literature [8–11, 19, 23, 32–43].

Second, four features that define IR studies were identified, and items for guiding equity considerations and analyses under each of these features were proposed to address gaps in existing guidance. The features include focus on implementation contexts, use of implementation strategies, inclusion of implementation outcomes, and use of IR design as part of an IR study or project [15]. The proposed items were informed by literature on IR and health equity; author’s own experience with clinical and public health practice and research in global health; teaching implementation research and health equity at the graduate level; and working with graduate students to analyze and operationalize concepts in IR and health equity.

Last, the proposed items were organized into a set of recommendations for prospective (planning, designing, and delivery of healthcare interventions) and retrospective analyses (assessment of ongoing or completed ESI implementation or IR studies). An inductive approach was applied to the proposed items to identify pathways by which they influence health equity when prospectively and/or retrospectively applied. Based on the pathways, implementation strategies for advancing health equity through IR in global health settings were identified.

## Summary of existing guidance on how to address health equity through IR in global health

Many frameworks explicitly link health equity to implementation research [8–11, 19, 23, 32–43], but only a few provide specific guidance on how to prospectively consider equity in IR [9–11, 19, 40–42], especially in LMIC settings [19]. For example, frameworks exist for exploring the role of social determinants of health in implementing and selecting implementation strategies to overcome equity-related implementation problems, such as Health Equity Implementation Framework [32, 33], Extension of RE-AIM framework [34], Health Equity Measurement Framework [35], EQ-DI framework [8], Equity consideration of PRISM and RE-AIM framework [36], and Health Equity and Sustainability [37]. Similarly, protocols on how to develop frameworks and tools for linking IR to health equity exist [38]. However, they do not necessarily provide explicit guidance on how such protocols may be used to guide IR for achieving equity in global health or guide retrospective analyses of the equity impact of completed IR studies in global health settings.

The EquIR framework provides an example of how to prospectively consider equity as part of implementation practice or research in LMIC settings [16]. It describes 6 steps which starts with the assessment of the health status of the populations and provides guidance on what to do during the planning, design, implementation, and evaluation phases of a program, including how to operationalize implementation outcomes for equity assessment [19]. However, this guidance may only be applied prospectively and not retrospectively; and is broad, without a clear distinction of how it differentially applies to either implementation research or practice.

Other practical guidance for prospectively integrating concepts of implementation science into health equity research exists [9, 11] – with recommendations on how to identify study population and setting, use implementation outcomes in intervention design, understand mechanisms of inequities, and identify and adapt implementation strategies to address inequities [9]. However, they do not provide recommendations for how such guidance could be retrospectively applied. There are also broad recommendations on how implementation science could better address equity issues – cutting across research, policy, practice, and capacity building [10]; and calls for using implementation strategies to address contextual factors that affect vulnerable populations, and measuring equity as an implementation outcome for certain interventions [39]. However, further guidance on how to implement these broad recommendations by specific audiences, e.g., research teams, and explication of specific steps that may be necessary in retrospective analyses in global health, are needed.

## How to leverage IR to address health equity in global health

### Incorporating health equity goals as part of IR in global health

To leverage IR to address equity in global health, health equity goals must be incorporated as part of the IR study or project objectives. Health equity is often framed from a ‘deficit’ perspective as health inequities, which are health inequalities that are unjust [44, 45]. Health inequalities that qualify as inequities are differences in health outcomes and determinants undergirded by unjust differences in social, structural, and systemic determinants of health (i.e., the conditions in which people live and work, and the structure and systems that determine such conditions) [45–47]. Examples of structural and systemic determinants of health will include systemic racism and gender bias that permeate health, educational, labor, and legal systems [47–49]. The unjust nature of these differences is defined based on notions of social justice and human rights in a given social context. For example, there are distributions of social, structural, and systemic determinants of health that would be deemed unjust based on a collective sense of what justice means or violations of human rights and/or civil rights laws in most societies [50]. Hence, incorporating health equity goals as part IR in global health must measure or acknowledge extant health inequalities; unpack differences in social, structural, and systemic determinants that may be linked to these inequalities [10] – along with the injustices that underlie these differences [31].

### A systematic approach for addressing health equity in IR in global health settings

Tables 1 and 2 propose explicit guidance for implementation teams to systematically address health equity as part of IR, prospectively and retrospectively – considering four features that define IR, i.e., implementation contexts, strategies, outcomes, and IR designs [15, 51]. The items mapped to the IR design are only applicable to research while items mapped to the other features will apply to both research and practice.

**Table 1:** PROSPECTIVE APPLICATION (planning, designing, and delivery of healthcare interventions)

Guidance*	How to apply	Example of relevant methods
Implementation contexts		
1. Define health problems by differences among groups defined by ‘equity variables’ (use equity variables e.g., ‘PROGRESS’ – place of residence, race/ethnicity, occupation, gender, religion, education, socioeconomic status, social capital and disability status [52–54] – to define groups and categorize their health and/or implementation problems with the evidence-supported intervention (ESI) of interest). Define groups that are socially disadvantaged based on the definition of health problems by different equity variables.	a. Determine how the burden of a health problem (e.g. incidence or prevalence of a vaccine-preventable disease) compares for groups categorized by relevant equity variables (e.g. race, socioeconomic status, gender, disability status). b. Determine how the implementation problem with the ESI (e.g. vaccine) differ for groups categorized by relevant equity variables. The implementation problem can be indicated by measures of access and quality (e.g. measures of effective coverage [55] related to the ESI comparing different groups. c. Determine if differences in social determinants of health (e.g. income and social protection, job/employment, affordable health services, good housing, and transportation) [56] for the relevant groups contribute to any disparity in health and implementation problems. d. Based on a-c, determine which groups are socially disadvantaged with respect to health and implementation problems linked to differences in their social determinants of health.	• Secondary data analysis of available quantitative data (including population-based survey, surveillance, administrative, burden of disease data, and health management information system records) • Literature review • Qualitative interviews
2. Identify and work with relevant stakeholders (give special attention to the perspectives of socially disadvantaged groups) in defining the problems of interest, solutions, and implementation arrangements.	a. Identify representatives of socially disadvantaged groups defined from step 1 above – and consult with them to systematically characterize the underlying causes of any disparity in health and implementation problems (i.e., find out from their perspective why the health/implementation problems occur) – and identify possible solutions. b. Systematically characterize the underlying causes of any disparity in health and implementation problems with other relevant actors (e.g. ESI implementers, other health services providers, decision-makers around the ESI at different levels) – and identify possible solutions. c. Compare perspectives (causes, causal pathways, and solutions) of different actors prioritizing instances where they agree, and the perspective of socially disadvantaged groups where there is no agreement.	• Stakeholder meetings and analyses [57] • Theory of change workshop and group modeling to capture a variety of causes, pathways, and potential solutions [58] • Root cause analysis techniques (e.g. ‘5 whys’ [59]) with specific groups for problems with limited number of causes and pathways • Other participatory methods [60] • Literature review
3. Unpack the social contexts and causal chain that may have contributed to health inequities (i.e., identify how specific structural and systemic determinants of health may have contributed to the health problems for socially disadvantaged groups), and propose mechanisms for health inequities (e.g. specify the type of social injustices and/or human rights violations).	a. Define the inner and outer context of implementation for the ESI. The inner context encompasses the characteristics of the beneficiaries of the ESI, implementers, implementing organizations (e.g. structure, climate), and implementation level (e.g. households, communities, health facilities). The outer context encompasses the sociopolitical, economic, and policy environment that are external to the inner context. [61, 62] b. Explicate any power differentials between actors within the inner context of implementation and arrangements for health services delivery (e.g. beneficiaries of ESI vs. implementers of ESI, researchers vs. research subjects, communities vs. implementing organization, health services providers vs. clients). c. Identify any specific social injustices and/or human rights violations within the inner context of implementation and arrangements for health services delivery (e.g. discrimination, oppression, exclusion from services based on group membership). d. Consider how historical antecedents (e.g., colonization, slavery, group supremacy) may have shaped advantages and disadvantages within the health system that governs the inner context of implementation and delivery of other health services. Consider how historical antecedents may have shaped other systems and structures (e.g. labor, housing, and transportation systems) that govern any pertinent social determinants of health identified from step 1. e. Identify how specific systemic and structural determinants of health [49] may have contributed to the disparity in health and implementation problems affecting socially disadvantaged groups (e.g. what role within relevant systems do institutionalized racism, [47] gender-based discrimination, classism [63] and ableism [64] contribute to the disparity in health and implementation problems affecting the socially disadvantaged groups?)	• Literature review • Qualitative interviews with all actors, including implementers and members of socially disadvantaged groups • Direct observations during stakeholder meetings • Participatory methods [60]
4. Adapt ESI and the inner context of implementation (implementing organization; implementation level e.g., households, communities, health facilities; implementers’ and beneficiaries' characteristics) to address implementation problems for socially disadvantaged groups.	a. Use a systematic approach involving relevant actors to make modifications to the ESI and inner context of implementation to address implementation problems (e.g. improve quality and access to the ESI) for the socially disadvantaged groups (e.g. change time and venue of vaccine delivery to when and where is convenient for members of the socially disadvantaged groups). The modifications could be guided by various implementation research (IR) adaptation frameworks and models. [65, 66] b. Specify strategies to address power differentials among actors within the inner context of the implementation (e.g. establish a social accountability mechanism [67] such as client feedback or community scorecard where implementers are held accountable to members of the socially disadvantaged groups). The strategy specification here (and subsequently) could be guided by various IR strategy frameworks. [68, 69] c. Test adaptations and strategies with members of the socially disadvantaged groups and make further modifications as necessary (e.g. conduct an initial pilot or qualitative assessment of the adaptations with members of the socially disadvantaged groups).	• Literature review • Participatory methods [60] • Focus groups and qualitative interviews • Theory-based analyses (application of IR theory, models, and frameworks) [70]
Implementation strategies**		
1. Include implementation strategies that target mechanisms of health inequities (e.g., addressing specific and relevant social injustices and human rights violations) within the inner context of implementation.	a. Consider and specify strategies for addressing social injustices and human rights violations within the inner context of implementation (e.g., establishing anti-discriminatory policies and safe procedures for reporting anti-discriminatory and oppressive practices, [71] enforcing existing antidiscrimination policies, [49] incentivizing and promoting practices that uphold rights of members of socially disadvantaged groups, and institutionalizing social accountability strategies). [67]	• Theory-based analyses (application of IR theory, models, and frameworks). [70]
2. Include implementation strategies that target structural and systemic determinants of health inequities within the inner context of implementation (and the outer context as much as feasible).	a. Consider and specify strategies for addressing pertinent social determinants of health relevant to the inner context of implementation, and the outer context as much as feasible (e.g. provision of subsidized or free access to good quality health services, income supplements such as conditional cash transfer, subsidized and good quality housing, transportation vouchers to socially disadvantaged groups). [72] b. Consider and specify strategies for addressing negative historical antecedents and pertinent structural and systemic determinants of health relevant to the inner context of implementation, and outer context as much as feasible (e.g. training and deploying tools for addressing unconscious bias and discrimination within health systems, [73] setting livable minimum wages and maternity leave with benefits within labor systems, affirmative actions and reparations throughout all systems. [49, 74]	• Theory-based analyses (application of IR theory, models, and frameworks) [70]
Implementation outcomes		
1. Prioritize implementation outcomes for the socially disadvantaged groups.	a. Operationally define and measure relevant implementation outcomes (IO) [75, 76] e.g., acceptability and fidelity linked to the adapted ESI and any specified implementation strategies from the perspectives of socially disadvantaged groups, *i.e. primary IO*. Consider taking measurements of these IO at more than one time point. b. Operationally define and measure other relevant IO (e.g. uptake and sustainment of the ESI) for all groups (encompassing both socially and disadvantaged and advantaged groups), *i.e. secondary IO* – and compare measures for socially disadvantaged groups vs. advantaged groups. Consider taking measurements at more than one time point. c. Link any differences in the secondary IO (e.g. uptake) comparing disadvantaged groups vs. advantaged groups to changes in the primary IO (e.g. acceptability) for disadvantaged groups.	• Theory-based analyses (application of IR theory, models, and frameworks) [70] • Psychometric approaches to develop and adapt tools for quantitative assessment • Mixed [77] and multi-methods [78] (combining various methods as needed along the research process – and not necessarily combining quantitative and qualitative methods for a single set of hypotheses as observed in mixed methods)
IR design		
1. Use pragmatic research designs to generate evidence for implementation (impact of design features on implementation outcomes and overall health outcomes for all population and the socially disadvantaged groups).	Consider the following research objectives: a. Include a research objective to estimate changes in implementation outcomes (IO) for all populations and the socially disadvantaged groups. b. Include a research objective to estimate changes in the overall health outcomes (e.g. morbidity and mortality) for all populations and socially disadvantaged groups. c. Examine time varying changes in implementation outcomes linked to changes in overall health outcomes for all populations and socially disadvantaged groups. d. Examine time varying changes in implementation outcomes comparing socially disadvantaged vs. advantaged groups linked to time varying changes in overall health outcomes comparing socially disadvantaged vs. advantaged groups.	• Quantitative IR study design [79, 80] • Qualitative IR study design [81] • Mixed [77] and multi-methods [78] (combining various methods as needed along the research process – and not necessarily combining quantitative and qualitative methods for a single set of hypotheses as observed in mixed methods) • System science methods [82]

^*^ The guidance described here can be applied as a whole or in part depending on which IR cardinal features (e.g. implementation context, strategies, outcomes, and IR design) is incorporated in the implementation research or practice project. Similarly, the considerations under each IR feature can be applied as a whole or in part depending on what is feasible for the implementation team

**Table 2:** Retrospective Application (assessment of ongoing or completed ESI implementation or IR studies)

Key Question*	How to apply	Example of relevant methods
*Six key questions:*		
Implementation context		
1. Who is benefiting most from the ESI and implementation strategies (beneficiaries) or carrying most of the burden of the health problem?	a. Review burden of disease and project data. Consider disaggregating magnitude and/or severity of a health problem and benefits of ESI and implementation strategies by relevant equity variables (e.g., ‘PROGRESS’—place of residence, race/ethnicity, occupation, gender, religion, education, socioeconomic status, social capital, and disability status). b. Examine the quantity and quality of the ESI and implementation strategies received by different relevant social groups. c. Explore the programmatic experiences (including any implementation problems) of members of different relevant social groups with the ESI and implementation strategies. Highlight the experiences of socially disadvantaged groups relative to the advantaged groups. d. Track and review data on implementation processes. Highlight any differences in the implementation procedure for socially disadvantaged groups compared to the advantaged groups – and whether these differences contribute to any negative experiences with the ESI and implementation strategies reported by the socially disadvantaged groups.	• Secondary data analysis of available quantitative data (including population-based survey, surveillance, administrative, burden of disease data, and health management information system records) • Qualitative methods (including interviews, direct observations, and focus group discussions) • Process documentation • Document review
2. What is the power differential between implementers and beneficiaries of the ESI and strategies – who is accountable (‘voice and teeth’) to who? Similarly, what is the power differential between researchers and research subjects in IR – who is accountable (‘voice and teeth’) to who?	a. Interrogate processes for decision-making and priority setting, resource administration, and accountability within the project, and compare the influence of key actors in these processes e.g. implementers vs. beneficiaries, socially disadvantaged vs. socially advantaged beneficiaries, researchers vs. research subjects. b. Examine whether beneficiaries or research subjects know and can implement mechanisms for holding implementers or researchers accountable, e.g. boycotting services or withdrawing participation (‘exit’), influencing resource administration.	• Qualitative methods (including interviews, direct observations, and focus group discussions)
3. Whose ethics, values, and knowledge of evidence and how this is produced (epistemology) guided the description of the health problem and/or decision, selection, and implementation of the ESI?	a. Interrogate whether any participatory approaches were employed in the process for decision-making and priority setting on health problems, selection of the ESI and any implementation strategies, resource administration, and accountability within the project, and compare the influence of key actors in these participatory processes e.g. implementers vs. beneficiaries, socially disadvantaged vs. socially advantaged beneficiaries, researchers vs. research subjects. b. Examine whether local and indigenous knowledge and custom were sought and considered in the decision-making on health problems and causes, ESI/implementation strategies and their supporting evidence. c. Clarify how the local and indigenous knowledge and custom were employed in the implementation. Explore specific leadership, administrative and/or advisory roles of community duty-bearers and members of socially disadvantaged groups in the project implementation.	• Qualitative methods (including interviews, direct observations, and focus group discussions)
Implementation strategies		
4. Were any structural and/or systemic determinants of health addressed in implementation? How?	a. Examine whether any specific implementation strategies were employed to address social injustices and human rights violations within the inner context of implementation (e.g., establishing and enforcing anti-discriminatory policies). [49] b. Examine whether any specific implementation strategies were employed to address pertinent social determinants of health (e.g. provision of subsidized or free access to good quality health services). [72] c. Examine whether any specific implementation strategies were employed to address negative historical antecedents, structural and systemic determinants of health (e.g. training and deploying tools for addressing unconscious bias and discrimination within health systems). [73]	• Process documentation • Document review
Implementation outcomes		
5. Was any evaluation of the implementation project completed? Whose outcomes (implementation and health outcomes) served as the basis for the evaluation or are being monitored (for ongoing implementation)?	a. Examine whether an evaluation was completed for the project, and if any specific implementation outcomes (e.g. acceptability, uptake) were defined from the perspective of socially disadvantaged groups as part of the evaluation.	• Process documentation • Document review
IR design		
6. Was a pragmatic research design used to generate evidence for the implementation to include impact of the design features on implementation outcomes and overall health outcomes for all populations and socially disadvantaged groups?	a. Examine the specific IR design that was used to generate evidence for implementation. b. Explore if any differential impact of the ESI and/or implementation strategies were examined on implementation and health outcomes for socially disadvantaged vs. advantaged groups.	• Process documentation • Document review

^*^ The key questions described here can be applied as a whole or in part depending on which IR cardinal features (e.g. implementation context, strategies, outcomes, and IR design) are incorporated in implementation research or practice project

For all prospective IR studies or projects, an analysis of health inequalities surrounding health problems and implementation challenges that form the focus of the study or project could be included as part of the description of the implementation context – and evidentially linked to any unjust differences in the structural and systemic determinants of health (e.g., how institutional racism or gender bias influences a specific health problem or implementation challenge) to qualify the inequalities as inequities (Table 1). Similarly, implementation strategies could be packaged to address power differentials among relevant actors and pertinent structural and systemic determinants of health in specific settings – irrespective of whether equity is the primary research question or project’s objective. Strategies targeting the structural and systemic determinants of health inequities should be conceptualized as distinct from adaptations to the ESI or inner contexts of implementation to improve access to the ESI (or other health services) for socially disadvantaged groups (Table 1).

For completed IR studies or implementation projects, researchers can retrospectively evaluate how decisions were made on the ESI and implementation strategies and how the power play among the different actors involved (Table 2) – and whether the beneficiaries participated in the decision (‘voice’) and had means to hold implementers accountable (‘teeth’) during the implementation process [67, 83]. Such retrospective analyses may also be embedded within prospective studies to examine how historical antecedents may have shaped health problems and implementation challenges that will be studied, and to guide efforts to mitigate these influences as much as possible.

### Pathways by which equity considerations in IR impact health equity in global health settings

**Fig. 1:**
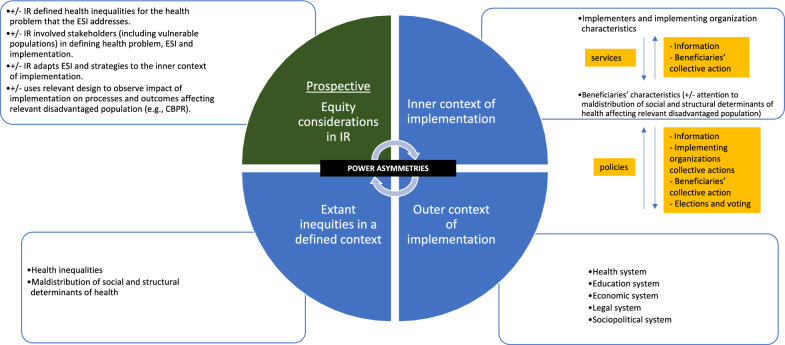
Prospective Application of IR for Health Equity

**Fig. 2:**
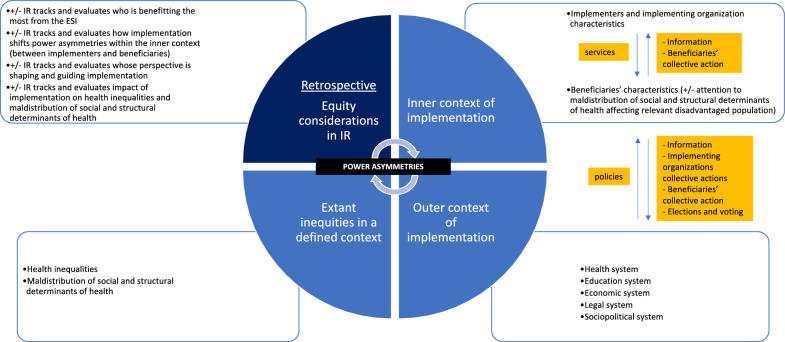
Retrospective Application of IR for Health Equity

Figures 1 & 2 describe pathways for understanding how IR studies or projects could impact health equity when the considerations from Tables 1 and 2 are applied. Central to these pathways is the recognition that power, i.e., “the ability to shape the thinking and/or actions of other actors” [84], is never uniformly distributed among actors [85]. That is, power is fluid and asymmetric among different actors (e.g., socially advantaged vs. disadvantaged populations, implementers vs. beneficiaries, researchers vs. research subjects, government officials vs. citizens) – and these asymmetries govern the implementation context that shapes how the implementation of ESI or IR studies influences the extant health inequities in a given setting. The proposed equity considerations operate by precipitating power negotiations among actors and shifting the balance of power to directly influence the inner context of implementation, and indirectly the outer context. Both the inner and outer context of implementation in turn shape the unjust distribution of health determinants and outcomes that is conceptualized as health inequities.

For example, a prospective IR study (Fig. 1) that defines unjust health inequalities and packages implementation strategies to address these differences may involve alterations to the service delivery arrangements based on feedback from socially disadvantaged groups or their boycott of services (‘exit’). The mechanism by which feedback or exit by socially disadvantaged groups influences the service delivery arrangements by implementers involves shift in the balance of power which will ultimately affect the inner context of implementation. The same mechanism if enacted at scale (e.g., feedback or exit by a large coalition of socially disadvantaged groups) may influence implementers to organize and influence government and policies (e.g., through lobbying activities), and future elections. Thus, the collective actions from socially disadvantaged groups, allies, and implementers may influence policies and systems, i.e. the outer context, that govern the production of the intended services through a negotiation of power distribution.

The same analogy is true for a retrospective analysis (Fig. 2), the only difference is the timing of the analysis relative to the health inequities in question. In this case, the equity consideration, e.g., how decisions were made on the ESI and implementation strategies and the power play among the different actors involved, would have directly shifted the balance of power within the inner context and between relevant actors at some point in the past – and influenced the power dynamics within the outer context if organized at scale.

### Balancing power asymmetries in IR to advance equity in global health

Power asymmetries, i.e., differences in power between advantaged and disadvantaged groups (e.g. HIC vs. LMIC entities), [86] have been previously identified as a critical component of global health governance [87], but was not explicitly linked to implementation efforts or unraveling pathways by which implementation efforts or IR contribute to health equity in global health. Indeed, from the initial decision-making on a problem or ESI, through the appropriation of benefits and risks that accrue from the implementation activities, power is constantly negotiated among the different actors at different stages [46].

Implementers linked to organizations originating from HIC may hold more structural power (power related to knowledge and technical know-how) [84] over beneficiaries of ESI in LMIC. The way that this structural power is negotiated or wielded within the inner context of implementation can influence health equity both directly and/or indirectly. Other implementation efforts may attract significant financial (money and asset) and discursive (ability to shape how a subject is discussed) powers that are sufficient to sway the dynamics of operations within government in some LMIC – and thus extend the influence of the implementation efforts outside of the inner context of implementation. Hence, by focusing attention on power asymmetries, and itemizing equity considerations for highlighting these asymmetries, implementation strategies can be packaged to target the balance of power as part of implementation of ESI or IR studies.

### Implementation strategies to balance power asymmetries in IR to advance equity in global health

Implementation strategies for balancing power asymmetries within the inner context may involve training in diplomacy, i.e., the art and practice of conducting negotiations [88], for relevant actors involved in implementation efforts, including community representatives [18, 89]. Other strategies could involve power sharing, e.g., formulation of representative and engaged community or advisory boards to guide decisions on key implementation activities [90], creating opportunities for representatives of disadvantaged groups or their advocates to lead implementation effort (or relevant aspects) where feasible [91], and participatory monitoring and evaluation (e.g., use of community scorecard) for social accountability [92, 93]. Power sharing does not necessarily translate to equal power but involves steps to intentionally include all parties along the power asymmetries, and creating a respectful and genuine platform for each party to participate in decisions around the implementation efforts.

Further, implementation strategies could be tailored to address certain structural and systemic determinants of health and social injustices that operate within the inner context of implementation. For example, training and tools to address unconscious bias and discrimination among implementers [73], enforcing anti-discriminatory policies [49], and using results from participatory monitoring and evaluation activities to inform structural changes that address discrimination, oppression, and exclusion within the services delivery arrangement [91].

There are other strategies to consider for tackling the root causes of health inequities within the outer context more broadly [49, 72–74]. Strategies targeting the social determinants of health may include provision of subsidized or free access to good quality health services (other than the ESI), supplemental income, good quality housing, and transportation vouchers [72]. Strategies targeting the structural and systemic determinants of health (and their historical antecedents) may include eliminating discriminatory practices in health system, setting livable minimum wages in labor system, affirmative actions and reparations throughout all systems [49, 73, 74]. The implementation of some of these strategies call for multisectoral approaches [94]. Thus, implementation teams should seek opportunities to join efforts with other relevant entities in enacting them as practically possible.

## Conclusions

Implementation of ESI and IR studies provides an avenue for achieving global health goals. However, explicit considerations for health equity as part of implementation research and practice are needed for this potential to be realized. Such explicit considerations should look back as much as possible, and entail defining and analyzing health inequities and intervening on the underlying causes and mechanisms of health inequities as part of IR on a routine basis. Implementation strategies targeting these causes and mechanisms mainly operate by shifting the balance of power among different actors involved in the implementation of ESI and IR studies especially within the inner context of implementation.

## Data Availability

Data sharing is not applicable to this article as no datasets were generated or analyzed.

## Abbreviations

ESI: Evidence-supported health interventions
HIC: High-income countries
IR: Implementation research
IS: Implementation science
LMIC: Low- and middle-income countries
PROGRESS: Place of residence, race, occupation, gender, religion, education, socioeconomic status, social capital
TMF: Theories, models and frameworks
